# Association between hemoglobin trajectories and the incidence of dementia in a cohort of females aged 55–79 years

**DOI:** 10.1371/journal.pone.0300784

**Published:** 2024-04-03

**Authors:** Dong Yoon Lee, Jihyeon Jeong, Won-Il Choi

**Affiliations:** 1 Department of Internal Medicine, Myongji Hospital, Hanyang University, Goyang, South Korea; 2 Department of Statistics, Kyungpook University, Daegu, South Korea; University of Haifa, ISRAEL

## Abstract

**Purpose:**

To assess the association between pattern changes in hemoglobin levels over time and the incidence of dementia using trajectories in females aged 55–79 years.

**Materials and methods:**

We conducted a retrospective cohort study using females of aged 55–79 years from the National Health Insurance Service National Health Screening Cohort in Korea. To identify hemoglobin trajectories over eight years (2002–2009), we performed a three-step approach comprising measures of change, factor analysis, and cluster analysis. Univariate and multivariate Cox proportional hazard models were used to assess the associations between hemoglobin trajectories and the incidence of dementia.

**Results:**

We included 20,195 of 235,742 female participants. New dementia (N = 2664) was developed during follow-up period (2010–2015). Five hemoglobin trajectories were identified: high, mid, low, increasing, and decreasing. With high as a reference, the hazard ratios (HRs) for low and decreasing trajectories were significant, 1.28 (95% confidence interval [CI], 1.13–1.45) and 1.21 (95% CI, 1.10–1.34) in univariate models, respectively. However, only the HR for the decreasing trajectory was significant, 1.12 (95% CI, 1.01–1.24) after adjustment for confounders.

**Conclusion:**

The decreasing trajectory of hemoglobin levels within the normal range was associated with dementia. Even females aged 55–79 years without anemia might be vulnerable to dementia development risk.

## Introduction

Dementia is one of the most prevalent progressive diseases in the world. Dementia prevalence was approximately 47 and 57.4 million worldwide in 2015 and 2019, respectively [1,2]. Its prevalence is expected to increase to 152.8 million by 2050, particularly females who were shown susceptible to the risk of dementia than males [2]. According to a meta-analysis study in Korea, the prevalence of dementia among those 65 years or older was 9.2%, which was shown to be higher than that in any other Western or Asian country. Specifically, dementia prevalence was 10.7% in females compared with 6.8% in males [3].

Many studies have been conducted on the risk factors that might facilitate the development of dementia [4–8]. Of them, anemia (hemoglobin levels <12.0 g/dL in females) with or without iron, folate, and vitamin B12 deficiency, may play an important role in cognitive health including dementia [9–14]. A National Health Insurance Service cohort study in Korea showed a dose-response relationship of incident dementia according to the severity of anemia [15]. Nonlinear, low, and high hemoglobin levels and anemia subtypes were assessed on the risk of dementia or cognitive function [16,17]. However, these studies might not fully elucidate the association of dementia with anemia. The fact that hemoglobin levels change in patterns over time has been often neglected without applying proper analysis to reflect the pattern change of the hemoglobin. To examine the longitudinal trajectory types of hemoglobin levels and dementia, one previous study performed a group-based trajectory modeling (GBTM) approach [18]. They used the small-town community-dwelling older Japanese with rather small participants. Applying a GBTM approach in observational studies may overestimate the risk due to limited criteria and study design [19]. There could be approaches to assess the longitudinal trajectories other than a GBTM [20,21]; for instance, the three-step approach comprises of measures of change, factor analysis, and cluster analysis [22].

To our knowledge, this is the first study to apply a three-step approach to investigate the association between the longitudinal trajectories of hemoglobin levels and dementia. We assessed the association between the trajectories of hemoglobin levels over time and the incidence of dementia in females aged 55–79 years using large representative participants.

## Materials and methods

### Study population

**Fig 1:**
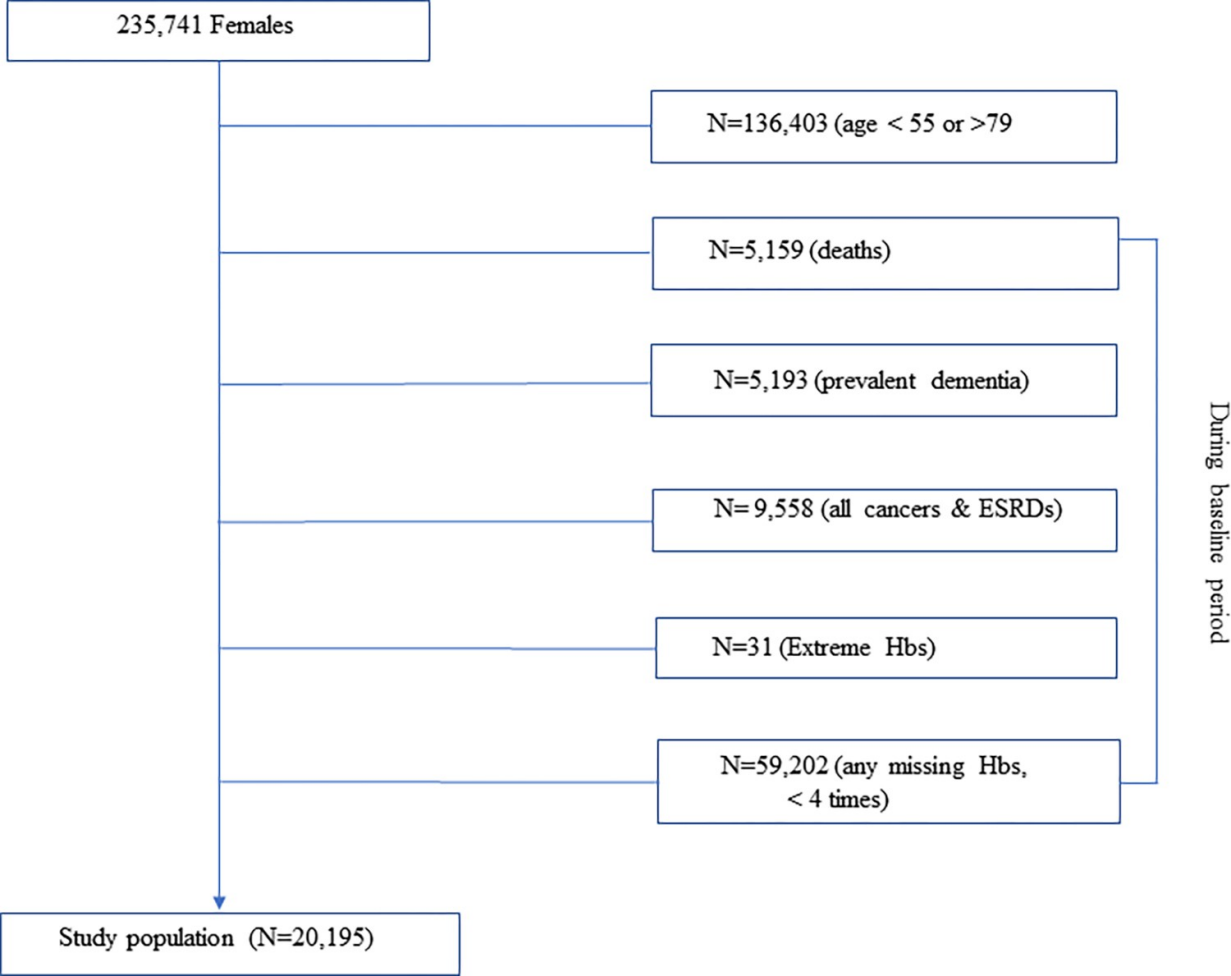
The flow diagram of the study population. Abbreviations: ESRDs, end-stage renal diseases; Hbs, hemoglobins.

We used the National Health Insurance Service National Health Screening Cohort (NHIS-HEALS) provided by the NHIS in the Republic of Korea (Korea). The details of the cohort profile have been published elsewhere [23]. Briefly, NHIS-HEALS was constructed to detect and prevent chronic diseases by making the database publicly available. The general health screening program can be applied at least once every two years for the entire population of Korean adults ≥40 years old, starting in 1995. In the eligible population, the participation rate was 74.8% in 2014. We excluded participants out of 235,741 females in the following sequences: <55 and >79 years old (N = 136,403), deaths (N = 5159), prevalent dementia (N = 5193), all cancers and end-stage renal diseases (N = 9558), extreme hemoglobin values (N = 31, <6.0 and >19.9 g/dL), and any missing hemoglobin values that were collected <4 times (N = 59,202). The final number of participants included in the analysis was 20,195 (Fig 1). We used females aged 55–79 years in whom the prevalence of dementia is high and change in hemoglobin levels should be less affected by menopause. We did not use records of participants who were >79 years old because of the high proportion of missing hemoglobin values. Baseline examinations were conducted in 2002 and 2003, and participants ≥40 years old were biennially screened and followed up until 2015.

Information on death (date and cause) from Statistics Korea was individually linked using unique personal identification numbers. By law, all deaths must be reported to Statistics Korea. The Myongji Hospital Institutional Review Board approved the current study (IRB number: MJH 2022-04-033), and the requirement for informed consent was waived because data in the NHIS database were anonymized in accordance with strict confidentiality guidelines. The present study followed the Strengthening the Reporting of Observational Studies in Epidemiology (STROBE) reporting guideline for cohort studies [24].

### Exposures

We used each 2 years of hemoglobin levels over 8 years (T1 2002–2003, T2 2004–2005, T3 2006–2007, T4 2008–2009). The hemoglobin measurement was performed as a part of the multiphasic blood screening examinations. Hemoglobin was measured by the cyanmethemoglobin method, which is the method of choice. Blood was collected at different healthcare centers.

### Outcomes

The follow-up period was six years between January 1, 2010 and December 31, 2015. We defined the newly developed dementia according to the International Classification of Diseases (ICD)-10 codes as follows: F00 (dementia in Alzheimer’s disease), F01 (vascular dementia), F02 (dementia in other diseases classified elsewhere), F03 (unspecified dementia), and G30 (Alzheimer’s disease). The first day of entry into a primary or secondary diagnosis was defined as the date of occurrence. Although it was not included in the diagnosis code, the date of death was counted as the onset date if the cause of death during follow-up was dementia.

### Potential confounders

The potential confounders included age groups (55–59, 60–69, 70–79), residential area (Seoul the capital city, large metropolitan cities, others), household income (monthly insurance contributions relative to the median, a proxy measure for income) (90–100%, 60–89%, 30–59%, 10–29%, 0%), smoking status (never/ever/current), drinking (rarely, 2–3 times/month, 1+/week, 3+/week), physical activity (none, 1–2 times/week, 3–4 times/week, 5+ times/week), body mass index (BMI) (<23.0 kg/m^2^ and ≥23.0 kg/m^2^), and chronic diseases such as hypertension (yes/no) and diabetes (yes/no). Data on smoking status, drinking, and physical activity were obtained using a questionnaire. Smoking status was categorized as never/ever/current-smoker. Drinking and physical activity were categorized according to the frequency per week. The BMI was calculated using weight (kg) divided by height in meters squared (m^2^). The cutoff value of the BMI (23 kg/m^2^ is the intermediate cut-off point) was categorized for Asian populations according to WPRO WHO’s recommendation [25,26]. Hypertension and diabetes were defined using diagnostic codes, self-reports (yes/no; “Have you been diagnosed with or taken medication for any of the following diseases?”), or laboratory test results. For laboratory tests, hypertension was defined as a systolic blood pressure ≥140 mmHg or diastolic blood pressure ≥90 mmHg. Diabetes was defined as a glucose level of ≥126 mg/dL.

### Statistical analysis

Continuous variables are presented as mean ± standard deviation (SD), and categorical variables are presented as frequencies and percentages. Student’s t-test for continuous variables and the chi-squared test for categorical variables were performed to compare differences between dementia and non-dementia.

To identify pattern changes in hemoglobin trajectories over time, we performed a three-step approach proposed by Leffondre et al. (2004) [22]. Measures of change over time were originally 27. These measures were divided into five main categories as follows: basic descriptive statistics, elementary measures of change, measures of nonlinearity and inconsistency of changes, measures sensitive to nonmonotonicity and abrupt short-term fluctuations, and measures contrasting early vs. later change. The measures measure the relative importance of the major abrupt change and the measures contrasting early vs. later change were measures 17, 18, 25–27, which were excluded because the extreme values of the hemoglobin were excluded in this study. First, we calculated measures to ascertain the changes in trajectory over time. Second, among measures of change, we performed factor analysis a variable reduction to select appropriate measures of change. Factors were extracted using principal component analysis, and the number of factors was determined using a scree plot [27]. Rotated factor loadings with varimax were used. We selected measures whose loadings were high and easy to interpret. Finally, K-means cluster analysis was used to classify the participants according to their multivariate pattern of change represented by the measures selected using factor analysis [28]. The number of clusters must be fixed a priori. Three criteria were used for the number of clusters: observed overall R2, pseudo-F statistic, and cubic clustering criteria (CCC). Observed overall R2 is the ratio of the between-clusters variance to the total variance. Pseudo-F statistic is the ratio of between-clusters mean square to within-clusters mean square. CCC compares the observed overall R^2^ to the approximate expected R^2^ [22]. Cox proportional hazard models were used to assess the association between hemoglobin trajectories and the incidence of dementia. Crude and adjusted analyses were performed with and without adjustment for age groups, household income, residential area, BMI, smoking status, drinking, physical activity, hypertension, and diabetes. Data manipulation from the databases and statistical analyses including factor and cluster analyses were performed using SAS software, version 9.4 (SAS Institute Inc., Cary, NC, USA). The calculation of measures of change was performed by using the ‘traj’ package in R software, version 3.3.3 (R Project for Statistical Computing, Vienna, Austria), and the Cox proportional hazard models were performed using SPSS Statistics for Windows, version 25 (IBM Corp., Armonk, NY, USA).

## Results

Participants who developed dementia during follow-up period were 2664, with an annual average incidence of 2198.6/100,000. The mean follow-up duration was 66.8 months (SD 14.5). The distribution of BMI, smoking, and drinking did not differ between dementia and non-dementia groups. Among individuals between 60–69 years of age who developed dementia, the incidence of dementia was higher in areas other than the capital city and large metropolitan residents who had no dementia. Moreover, household income in the group with dementia was higher when it came to comparison with the median. The incidence of dementia was lower when physical activity was performed more frequently. The incidence of dementia was higher in hypertension and diabetes groups. Hemoglobin levels were lower in the group with dementia compared to the non-dementia group (Table 1). Other descriptive statistics including median, quartile 1, and quartile 3 were shown comparable in both groups (S1 Table).

**Table 1:** Descriptive statistics of study variables by dementia.

Characteristics	Dementia (N = 2664)	Non-dementia (N = 17,531)	*P*-value^1^
Age group (years)			< 0.001
55–59	410 (15.4)	7793 (44.5)	
60–69	1619 (60.8)	8346 (47.6)	
70–79	635 (23.8)	1392 (7.9)	
Residential area			<0.001
Seoul, the capital city	274 (10.3)	2703 (15.4)	
Large metropolitan cities	569 (21.4)	4323 (24.7)	
Others	1821 (68.3)	10,505 (59.9)	
Household income (%)			0.02
90–100	803 (30.1)	5117 (29.2)	
60–89	728 (27.3)	5122 (29.2)	
30–59	623 (23.4)	4303 (24.5)	
10–29	509 (19.1)	2984 (17.0)	
0–9	1 (0.1)	5 (0.1)	
Body mass index^2^ (kg/m^2^)			0.05
<23.0	917 (34.4)	5745 (32.8)	
≥23.0	1742 (65.4)	11,772 (67.1)	
Smoking status^2^			0.42
Never	2484 (93.2)	16,433 (93.7)	
Ex	24 (0.9)	128 (0.7)	
Current	62 (2.3)	339 (2.0)	
Drinking^2^			0.71
Rarely	2324 (87.2)	15,224 (86.8)	
2–3 times/month	155 (5.8)	1142 (6.5)	
1+/week	70 (2.6)	446 (2.5)	
3+/week	36 (1.4)	220 (1.3)	
Physical activity^2^			<0.001
None	1889 (70.9)	11,308 (64.5)	
1–2 times/week	287 (10.8)	2629 (15.0)	
3–4 times/week	143 (5.4)	1163 (6.6)	
5+ times/week	276 (10.4)	1985 (11.3)	
Hypertension			<0.001
Yes	2065 (77.5)	12,019 (68.6)	
No	599 (22.5)	5512 (31.4)	
Diabetes			<0.001
Yes	1204 (45.2)	6208 (35.4)	
No	1460 (54.8)	11,323 (64.6)	
Hemoglobin (mg/dL)			
T1 (2002–2003)	12.95 ± 1.06	13.01 ± 1.01	<0.001
T2 (2004–2005)	12.84 ± 1.06	12.94 ± 1.01	<0.001
T3 (2006–2007)	12.87 ± 1.08	12.95 ± 1.03	<0.001
T4 (2008–2009)	12.74 ±1.12	12.88 ±1.06	<0.001

^1^Statistically significant based on *p* < 0.05.

^2^Missing number of participants: Body mass index (N = 5, N = 14); smoking status (N = 94, N = 631); drinking (N = 79, N = 499); physical activity (N = 69, N = 446).

Factor analysis was performed using the remaining 22 measures of change. For each factor, one measure among those that had the highest loadings, which balanced the formal criterion of factor loading with such pragmatic criteria as variability, interpretability, or logical relationships with other measures, was selected: m15 (maximum of the absolute first differences) for factor 1; m9 (slope of the linear model) for factor 2; m23 (ratio of the maximum absolute second difference to mean absolute first difference) for factor 3; and m2 (mean-over-time) for factor 4. The criteria (the scree plot and the interpretability and practicality) used by the factor analysis showed 4 factor solution is the most appropriate. These 4 factors together explained 88.5% of the total variance in the 22 measures (Table 2).

**Table 2:** Rotated factor loadings for the 22 measures of change.

Measures of change	Factor 1	Factor 2	Factor 3	Factor 4
Elementary measures of change				
M1 Range	0.95	-0.08	-0.23	0.03
M2 Mean-over-time	-0.05	0.02	0.04	0.96^1^
M3 SD	0.95	-0.08	-0.22	0.03
M4 CV	0.95	-0.09	-0.22	-0.09
M5 Change	-0.05	0.99	0.03	0.04
M6 Mean change per year	-0.05	0.99	0.03	0.04
M7 Change/first score	-0.01	0.99	-0.01	0.04
M8 Change/mean-over-time	-0.05	0.99	0.04	0.05
M9 Slope *b*	-0.05	0.97^1^	0.05	0.05
M10 R^2^ of the linear model	-0.16	-0.07	-0.74	-0.06
Measures of nonlinearity and inconsistency of change				
M11 Max Δ_1_	0.76	0.51	0.28	0.08
M12 SD Δ_1_	0.94	-0.01	0.30	0.06
M13 SD Δ_1_ per year	0.94	-0.01	0.30	0.06
M14 Mean |△_1_|	0.97	-0.03	0.13	0.07
M15 Max |△_1_|	0.97^1^	-0.05	0.10	0.03
M16 Max |△_1_|/mean-over-time	0.97	-0.05	0.09	-0.08
Measures sensitive to nonmonotonicity and to abrupt short-term fluctuations				
M19 Mean Δ2	-0.03	-0.04	0.03	-0.24
M20 Mean |Δ2|	0.85	-0.01	0.42	0.04
M21 Max |△2|	0.91	-0.01	0.38	0.05
M22 Max |△2|/mean-over-time	0.91	-0.01	0.37	-0.05
M23 Max |△2|/mean |△_1_|	0.12	0.03	0.86^1^	-0.07
M24 Mean |△2|/mean |△_1_|	0.10	0.02	0.85	-0.07

SD, standard deviation; CV, coefficient of variation. M11, Maximum of the first differences; M12, SD of the first differences; M13, SD of the first differences per time unit; M14, Mean of the absolute first differences; M15, Maximum of the absolute first differences; M16, Ratio of the maximum absolute first difference to the mean-over-time; M19, Mean of the second differences; M20, Mean of the absolute second differences; M21, Maximum of the absolute second differences; M22, Ratio of the maximum absolute second difference to the mean-over-time; M23, Ratio of the maximum absolute second difference to the mean absolute first difference; M24, Ratio of the mean absolute second difference to the mean absolute first difference.

**Fig 2:**
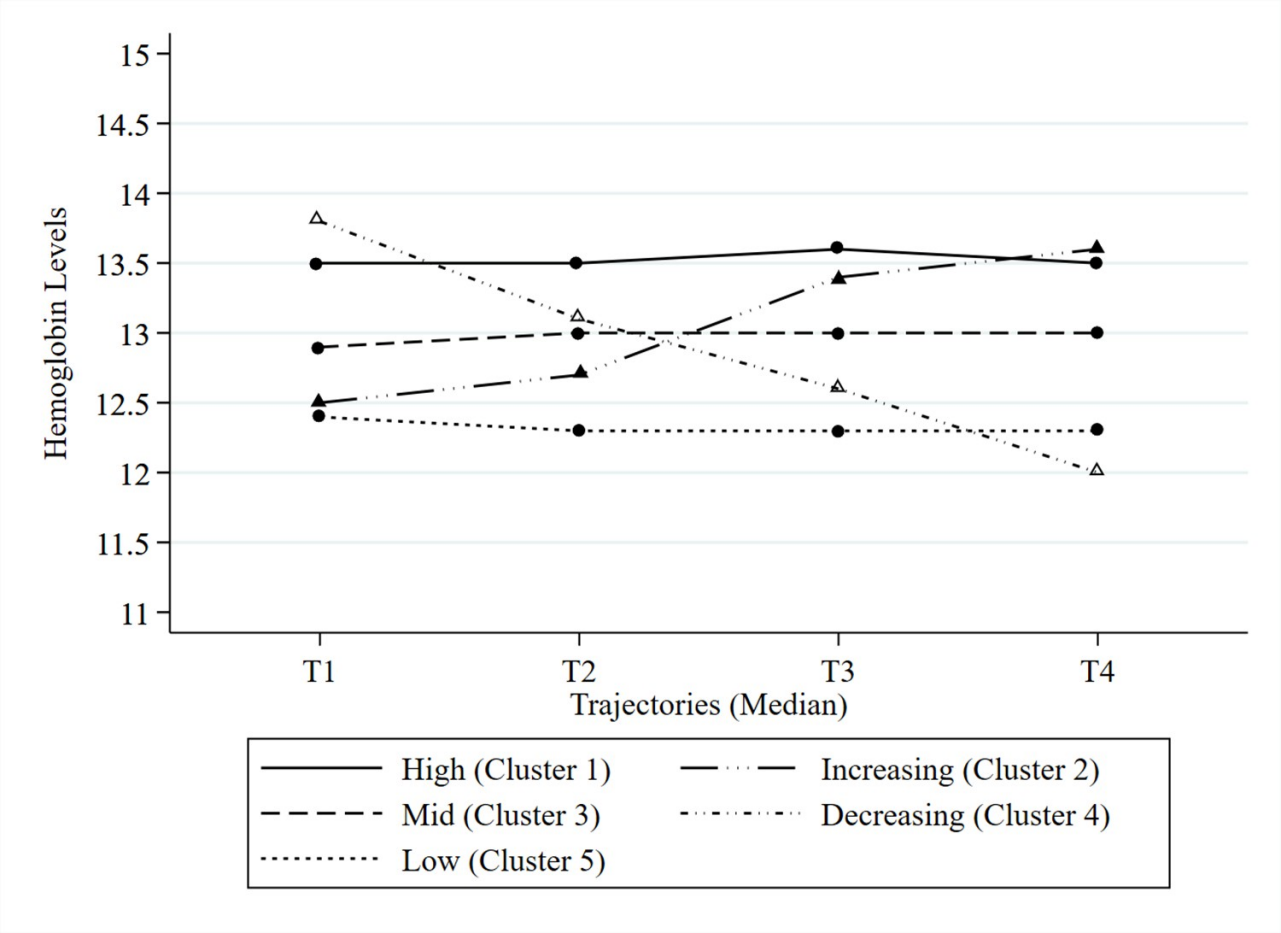
Hemoglobin trajectories by cluster. The graphs show the median hemoglobin measurements for each time frame of two years. T1 indicates 2002–2003, T2 indicates 2004–2005, T3 indicates 2006–2007, and T4 indicates 2008–2009.

Before performing cluster analysis with the four measures of change selected by the factor analysis, five clusters were determined according to the criteria, including pseudo-F statistics, observed overall R^2^, and the CCC (Table 3). The medians of the hemoglobin trajectories by cluster according to the four time periods (T1 2002–2003, T2 2004–2005, T3 2006–2007, and T4 2008–2009) were presented graphically. Five clusters were named as follows: high, mid, low, increasing, and decreasing. The trajectories were relatively stable and parallel over time, overlapping each other for high, mid, and low. The slopes of the increasing trajectories were slightly different over time. The slope for T2-T3 was steeper than T1-T2 and T3-T4, while that of the decreasing trajectory was relatively similar (S2 Table, Fig 2).

**Table 3:** Statistical criteria for the number of clusters.

	Number of clusters				
Criteria	2	3	4	5	6
Pseudo–F statistics	4137.28	3887.93	4077.67	4602.99	4632.54
Observed overall R^2^	0.18	0.33	0.45	0.55	0.60
CCC	–15.88	–42.25	–57.88	–64.09	–58.09

CCC, cubic clustering criterion.

Of the five trajectories, the low was the most common (29.3%), and increasing was the least common (11.1%). All study variables were significantly different among the five trajectories by the chi-square test. The high trajectory was the youngest, living in the capital, most engaged in physical activity, and had highest household income and most having a BMI of more than 23.0. Lifestyles such as smoking and drinking were not so different from other trajectories; however, hypertension and diabetes were different from decreasing trajectories. The decreasing trajectory was the oldest, living in areas other than the capital or large metropolitan cities, less engaged in physical activity, and had lowest household income and second-most having a BMI of more than 23.0; however, smoking and drinking were not different. As age was one of the strongest risk factors of dementia, decreasing trajectory was the oldest whereas high trajectory was the youngest. The low trajectory was the next oldest. The proportions of aged 70–79 years in decreasing, high, and low trajectory were 12.2%, 8.0%, and 11.4%, respectively (Table 4).

**Table 4:** Descriptive statistics of study variables by cluster.

Characteristics	High (n = 5756)	Mid (n = 3742)	Low (n = 5925)	Increasing (n = 2243)	Decreasing (n = 2529)
Age group (years)					
55–59	2631 (45.7)	1597 (42.7)	2226 (37.6)	876 (39.1)	873 (34.5)
60–69	2665 (46.3)	1804 (48.2)	3020 (51.0)	1129 (50.3)	1347 (53.3)
70–79	460 (8.0)	341 (9.1)	679 (11.4)	238 (10.6)	309 (12.2)
Residential area					
Seoul, the capital city	975 (16.9)	594 (15.9)	857 (14.5)	282 (12.6)	269 (10.6)
Large metropolitan cities	1416 (24.6)	972 (26.0)	1556 (26.2)	429 (19.1)	519 (20.5)
Others	3365 (58.5)	2176 (58.1)	3512 (59.3)	1532 (68.3)	1741 (68.9)
Household income (%)					
90–100	1794 (31.2)	1089 (29.1)	1800 (30.4)	556 (24.8)	681 (26.9)
60–89	1668 (29.0)	1098 (29.4)	1705 (28.8)	661 (29.5)	718 (28.4)
30–59	1362 (23.7)	914 (24.4)	1398 (23.6)	609 (27.1)	643 (25.4)
10–29	930 (16.1)	641 (17.1)	1019 (17.2)	417 (18.6)	486 (19.2)
0–9	2 (0)	0 (0)	3 (0)	0 (0)	1 (0)
Body mass index^1^ (kg/m^2^)					
<23.0	1586 (27.6)	1230 (32.9)	2296 (38.8)	737 (32.9)	813 (32.1)
≥23.0	4164 (72.3)	2511 (67.1)	3621 (61.1)	1504 (67.1)	1714 (67.8)
Smoking status^1^					
Never	5363 (93.2)	3505 (93.7)	5585 (94.3)	2093 (93.3)	2371 (93.8)
Ex	46 (0.8)	27 (0.7)	38 (0.6)	16 (0.7)	25 (1.0)
Current	126 (2.2)	70 (1.9)	86 (1.5)	55 (2.5)	64 (2.5)
Drinking^1^					
Rarely	4953 (86.0)	3255 (87.0)	5192 (87.6)	1956 (87.2)	2192 (86.7)
2–3 times/month	392 (6.8)	243 (6.5)	366 (6.2)	136 (6.1)	160 (6.3)
1+/week	166 (2.9)	90 (2.4)	135 (2.3)	56 (2.5)	69 (2.7)
3+/week	86 (1.5)	46 (1.2)	56 (0.9)	32 (1.4)	36 (1.4)
Physical activity^1^					
None	3580 (62.2)	2415 (64.5)	3915 (66.1)	1546 (68.9)	1741 (68.8)
1–2 times/week	928 (16.1)	536 (14.3)	848 (14.3)	291 (13.0)	313 (12.4)
3–4 times/week	409 (7.1)	271 (7.2)	343 (5.8)	129 (5.8)	154 (6.1)
5+ times/week	704 (12.2)	423 (11.3)	665 (11.2)	222 (9.9)	247 (9.8)
Hypertension					
Yes	4134 (71.8)	2569 (68.7)	3850 (65.0)	1619 (72.2)	1912 (75.6)
No	1622 (28.2)	1173 (31.3)	2075 (35.0)	624 (27.8)	617 (24.4)
Diabetes					
Yes	2162 (37.6)	1335 (35.7)	1995 (33.7)	826 (36.8)	1094 (43.3)
No	3594 (62.4)	2407 (64.3)	3930 (66.3)	1417 (63.2)	1435 (56.7)
Hemoglobin (mg/dL)					
T1 (2002–2003)	13.62 ± 0.69	12.89 ± 0.88	12.34 ± 0.67	12.5 ± 1.16	13.79 ± 1.05
T2 (2004–2005)	13.56 ± 0.74	12.94 ± 0.74	12.24 ± 0.72	12.83 ± 1.29	13.16 ± 1.18
T3 (2006–2007)	13.62 ± 0.73	12.98 ± 0.77	12.25 ± 0.73	13.35 ±1.28	12.61 ± 1.18
T4 (2008–2009)	13.57 ± 0.68	13.02 ± 0.92	12.17 ± 0.69	13.59 ± 1.05	11.98 ± 1.06

^1^ Missing number of participants: Body mass index (N = 6, N = 1, N = 8, N = 2, N = 2); smoking status (N = 221, N = 140, N = 216, N = 79, N = 69); drinking (N = 159, N = 108, N = 176, N = 63, N = 72); physical activity (N = 135, N = 97, N = 154, N = 55, N = 74).

The associations between the five trajectories of hemoglobin and the incidence of dementia were explored using Cox proportional hazard models. With high as a reference, low and decreasing trajectories showed statistically significant hazard ratios (HRs) of 1.28 (95% CI: 1.13–1.45) and 1.21 (95% CI: 1.10–1.34). Adjusted for confounding variables, the HR for the low trajectory was reduced to 1.06 with a loss of statistical significance. However, the HR for the decreasing was reduced to 1.12, which retained its significance (95% CI: 1.01–1.24). Potential confounders including age group, residential area, physical activity, hypertension, and diabetes were shown significant. The age group showed a large HR of 3.22–6.73 even after adjustments (Table 5).

**Table 5:** Hazard ratios and 95% confidence intervals of Cox proportional hazard models for incident dementia.

	Univariate^1^			Multivariate^2^		
Characteristics	HRs	95% CI	p-value^3^	HRs	95% CI	p-value^3^
Trajectory (ref. High)						
Mid	1.09	0.95–1.25	0.20	0.97	0.84–1.12	0.75
Low	1.28	1.13–1.45	< 0.001	1.06	0.93–1.21	0.34
Increasing	0.99	0.88–1.12	0.98	0.98	0.87–1.11	0.81
Decreasing	1.21	1.10–1.34	< 0.001	1.12	1.01–1.24	0.03
Age group (years) (ref. 55–59)						
60–69	3.51	3.15–3.91	< 0.001	3.22	2.88–3.61	<0.001
70–79	7.79	6.88–8.82	< 0.001	6.73	5.90–7.68	<0.001
Household income (%, ref. 90–100)						
60–89	0.91	0.82–1.00	0.07	0.99	0.90–1.10	0.97
30–59	0.92	0.83–1.03	0.16	0.98	0.88–1.10	0.84
10–29	1.08	0.97–1.21	0.14	1.05	0.93–1.17	0.40
0–9	1.13	0.15–8.04	0.90	0.82	0.11–5.89	0.85
Residential area (ref. Seoul)						
Large metropolitan	1.28	1.11–1.48	< 0.001	1.23	1.06–1.43	<0.001
Others	1.67	1.47–1.89	< 0.001	1.36	1.19–1.56	<0.001
Body mass index (kg/m^2^, ref. <23.0)						
≥23.0	0.92	0.85–1.00	0.06	0.93	0.85–1.01	0.11
Smoking status (ref. Never)						
Ex	1.23	0.82–1.84	0.30	0.98	0.63–1.51	0.93
Current	1.24	0.96–1.60	0.08	0.97	0.75–1.26	0.87
Drinking (ref. Rarely)						
2–3 times/month	0.88	0.75–1.04	0.15	1.00	0.85–1.19	0.91
1+ times/week	1.03	0.81–1.31	0.75	1.15	0.90–1.46	0.25
3+ times/week	1.07	0.77–1.48	0.68	1.13	0.81–1.58	0.43
Physical activity (ref. None)						
1–2 times/week	0.66	0.58–0.75	< 0.001	0.80	0.70–0.91	<0.001
3–4 times/week	0.74	0.62–0.88	< 0.001	1.02	0.86–1.22	0.74
5+ times/week	0.83	0.74–0.95	< 0.001	0.92	0.80–1.05	0.22
Hypertension during baseline (ref. No)						
Yes	1.55	1.41–1.70	< 0.001	1.19	1.08–1.31	<0.001
Diabetes during baseline (ref. No)						
Yes	1.46	1.36–1.58	< 0.001	1.32	1.22–1.43	<0.001

HR, Hazard ratio; CI, Confidence interval; ref, reference.

^1^Univariate model: Unadjusted.

^2^Multivariate model: Adjusted for age group, household income, residential area, BMI, smoking status, drinking, physical activity, hypertension, and diabetes.

^3^Statistically significant based on *p* < 0.05.

## Discussion

In the present study, we calculated 22 measures of change and conducted a factor analysis to select four measures as follows: maximum of the absolute first differences, the slope of the linear model, ratio of the maximum absolute second difference to mean absolute first difference, and mean-over-time. From these four measures, we were able to identify five distinctive hemoglobin trajectories (high, mid, low, increasing, and decreasing) by conducting a k-means cluster analysis. Adjusted for confounders the decreasing trajectory, not the low trajectory, had increased risk for dementia compared with the high trajectory.

To our knowledge, no direct comparison can be made because only one study assessed the association between the trajectory of hemoglobin and dementia, using a different statistical method and study population [18]. They applied the latent class GBTM approach to determine the trajectories of hemoglobin and albumin levels, identifying 3 trajectory patterns: high, moderate, and low. They demonstrated that the increased risk of dementia was associated with low trajectory hemoglobin levels compared with high trajectory. They used the Bayesian Information Criteria (BIC) and the Average Posterior Probability (APP) as the criteria. However, their three trajectories might inaccurately estimate the HR of dementia because relying on the BIC and the APP might have induced spurious subgroups of trajectory. Their study participants were rather small living in a small town.

We balanced the formal criterion of factor loading with such pragmatic criteria as variability, interpretability, or logical relationships with other measures [22]. Selection of measures for factors 3 and 4 based on the factor loadings was simple, however, selection of measures for factors 1 and 2 was rather arbitrary because many measures had overlapping high loadings. We selected measures of different domains, one of the measures within elementary measures for factor 2 and one of the measures within measures of nonlinearity and inconsistency for factor 1. The validity of our five trajectories could be supported in several ways. Five trajectories differed in the characteristics of the study participants. The decreasing trajectory was significantly associated with incident dementia even after adjustment of confounders compared with a high trajectory. It should be underscored that the decreasing trajectory over 8 years showed a decrease within the normal range of hemoglobin levels, and it was significantly associated with incident dementia among 5 trajectories. The different characteristics among trajectories and their association with the outcome may support the three-step approach. Further studies that apply various trajectory methods should be conducted to make a direct comparison with our study. Mesidor and colleagues recommended that the three-step approach can be performed as an alternative to compare with latent group-based trajectory modeling results [19].

Underlying mechanisms could be suggested relevant to the significant association between the decreasing trajectory of hemoglobin and incident dementia in females aged 55–79 years. First, decreased hemoglobin levels might induce hypoxia, which leads to cortical atrophy in various brain regions. The atrophy was shown significant in females [29]. A previous study that used the Rotterdam Study cohort demonstrated that low hemoglobin levels may worsen the structural connectivity and volume of white matter hyperintensities, which may indicate the risk of dementia [30]. Second, females who are in a transitional (from peri- to post-menopause) state might be more susceptible to endocrine aging [31]. Relevant to dementia, however, decreasing levels within the normal range over time is not studied; thus, further studies are needed.

### Strengths and limitations

Using the NHIS-HEALS which is a nationally representative cohort with a large number of participants, we observed a significant association between the decreasing trajectory of hemoglobin levels and the incidence of dementia. However, the monotonously low trajectory was not associated. Many of the previous studies on dementia or cognitive functions used a cut-off of WHO criteria for anemia [13,15,30,32,33]. The possibility of the risk of dementia within the range of normal levels may be neglected. We demonstrated that females aged 55–79 years without anemia might be vulnerable to dementia development risk because the decreasing trajectory within the normal range of hemoglobin level was associated with dementia. Above all, this is the first study to demonstrate a continuous pattern of change in hemoglobin levels, such as increasing and decreasing trajectories in association with the incidence of dementia. Increasing and decreasing trajectories can be more capable of estimating the risk of dementia in terms of biological plausibility compared with the previous study [18].

Nevertheless, our study has several limitations. First, our results might raise external validity issues because the study used data from Koreans only. Second, respondent bias due to the use of the questionnaire for social behavioral variables could be introduced. For instance, our study participants might be a cohort of females who lived in an environment in which smoking or drinking was considered socially undesirable. Third, the ascertainment of the new dementia from the health records may be incomplete. We defined dementia using the diagnosis codes without considering antidementia drugs. A cohort study using the NHIS-Senior comprised of participants over 60 years old showed a crude incidence of 1482.21 and 2696.31 in 2010 and 2015, respectively [34]. The annual average incidence of dementia in this study was 2198.6 per 100,000 during follow-up period (2010–2015). Fourth, the lack of standardization of hemoglobin value may influence our results because many medical institutions of various sizes participated in the national screening programs. Formal validity and reliability studies have not been performed. However, the advantages of the cyanmethemoglobin method are the easy standardization and stability of the reagent. Fifth, we could not include crucial potential confounders such as *APOE* ε4, hearing loss, and amyloid β because these were not part of the NHIS-HEALS cohort. Lastly, the selection of a parsimonious subset of measures of change of hemoglobin levels by factor analysis and trajectories by cluster analysis to group participants may be somewhat arbitrary despite of the proposed criteria.

### Conclusion

The three-step approach may be useful for identifying pattern changes in hemoglobin trajectories over time. Five distinctive trajectories (high, mid, low, increasing, and decreasing) were identified. Adjusted for confounding variables, the HR for the decreasing hemoglobin trajectory with high as a reference was 1.12 (95% CI: 1.01–1.24). The decreasing trajectories of hemoglobin levels within the normal range over time may be a warning sign for dementia in females aged 55–79 years.

## Supporting information

S1 Table Other descriptive statistics of hemoglobin levels by dementia. (DOCX)

S2 Table Medians of hemoglobin trajectories according to time frames. (DOCX)
