# Supplementary material for: Association between hemoglobin trajectories and the incidence of dementia in a cohort of females aged 55–79 years
Source: PLoS One. 2024 Apr 3;19(4):e0300784. doi: 10.1371/journal.pone.0300784 (PMC10990242; doi:10.1371/journal.pone.0300784)
Supplement: S1 Table — (DOCX) [file pone.0300784.s001.docx]

**S1 Table. Other descriptive statistics of hemoglobin levels by dementia.**

| **Characteristics** | **Dementia (N=2664)** | **Non-dementia (N=17,531)** |
| --- | --- | --- |
| **T1** |  |  |
| Median | 13.0 | 13.0 |
| Minimum | 6.0 | 6.4 |
| Maximum | 17.8 | 19.8 |
| Quartile 1 | 12.3 | 12.4 |
| Quartile 3 | 13.6 | 13.6 |
| **T2** |  |  |
| Median | 12.8 | 12.9 |
| Minimum | 6.1 | 7.3 |
| Maximum | 16.8 | 18.1 |
| Quartile 1 | 12.2 | 12.3 |
| Quartile 3 | 13.5 | 13.6 |
| **T3** |  |  |
| Median | 12.9 | 13.0 |
| Minimum | 8.0 | 6.8 |
| Maximum | 18.3 | 19.0 |
| Quartile 1 | 12.1 | 12.3 |
| Quartile 3 | 13.5 | 13.6 |
| **T4** |  |  |
| Median | 12.8 | 12.9 |
| Minimum | 6.9 | 6.1 |
| Maximum | 16.8 | 18.8 |
| Quartile 1 | 12.1 | 12.2 |
| Quartile 3 | 13.5 | 13.6 |

T1, 2002-2003; T2, 2004-2005; T3, 2006-2007; T4, 2008-2009.
