# Supplementary material for: Association between hemoglobin trajectories and the incidence of dementia in a cohort of females aged 55–79 years
Source: PLoS One. 2024 Apr 3;19(4):e0300784. doi: 10.1371/journal.pone.0300784 (PMC10990242; doi:10.1371/journal.pone.0300784)
Supplement: S2 Table — (DOCX) [file pone.0300784.s002.docx]

**S2 Table. Medians of hemoglobin trajectories according to time frames.**

| **Characteristics** | **T1** | **T2** | **T3** | **T4** |
| --- | --- | --- | --- | --- |
| Trajectory 1 (High) | 13.5 | 13.5 | 13.59961 | 13.5 |
| Trajectory 2 (Increasing) | 12.5 | 12.69922 | 13.39844 | 13.59961 |
| Trajectory 3 (Mid) | 12.89844 | 13 | 13 | 13 |
| Trajectory 4 (Decreasing) | 13.79883 | 13.09961 | 12.59961 | 12 |
| Trajectory 5 (Low) | 12.39844 | 12.29883 | 12.29883 | 12.29883 |

T1, 2002-2003; T2, 2004-2005; T3, 2006-2007; T4, 2008-2009.
